# Three order increase in scanning speed of space charge-controlled KTN deflector by eliminating electric field induced phase transition in nanodisordered KTN

**DOI:** 10.1038/srep33143

**Published:** 2016-09-09

**Authors:** Wenbin Zhu, Ju-Hung Chao, Chang-Jiang Chen, Shizhuo Yin, Robert C. Hoffman

**Affiliations:** 1 Department of Electric Engineering, Penn State University, University Park, PA 16802, USA; 2 Robert C. Hoffman, Army Research Laboratory, 2800 Powder Mill Rd., Adelphi, MD 20783, USA.

## Abstract

In this paper, we report a three orders-of-magnitude increase in the speed of a space-charge-controlled KTN beam deflector achieved by eliminating the electric field-induced phase transition (EFIPT) in a nanodisordered KTN crystal. Previously, to maximize the electro-optic effect, a KTN beam deflector was operated at a temperature slightly above the Curie temperature. The electric field could cause the KTN to undergo a phase transition from the paraelectric phase to the ferroelectric phase at this temperature, which causes the deflector to operate in the linear electro-optic regime. Since the deflection angle of the deflector is proportional to the space charge distribution but not the magnitude of the applied electric field, the scanning speed of the beam deflector is limited by the electron mobility within the KTN crystal. To overcome this speed limitation caused by the EFIPT, we propose to operate the deflector at a temperature above the critical end point. This results in a significant increase in the scanning speed from the microsecond to nanosecond regime, which represents a major technological advance in the field of fast speed beam scanners. This can be highly beneficial for many applications including high-speed imaging, broadband optical communications, and ultrafast laser display and printing.

The optical beam deflector is widely adopted in many fields including imaging, printing, sensing, displays, and telecommunications. Traditional mechanical beam deflectors, such as those employing galvanic mirrors, polygonal mirrors, and microelectromechanical system mirrors, have limited scanning speed. To achieve a higher scanning speed, beam deflection based on the electro-optic (EO) effect has been investigated. In comparison to other types of EO materials, such as KH_2_PO_4_^1^, PZT^2^, and LiTaO_3_^3^, potassium tantalate niobate (KTN) has the advantage of an exceptionally large quadratic EO coefficient^4^,^5^. With the advent in recent years of high quality sizable KTN crystals that are suitable for device fabrication, there has been a growing interest in KTN crystals for different applications such as EO modulators, dynamic optical waveguides, and high-speed beam scanners^6^,^7^,^8^,^9^,^10^,^11^,^12^. The unique features of high scanning speed and non-mechanical movement make the KTN crystal-based beam scanner useful in many different optical systems, such as high-speed spectrometers^13^, high speed optical coherent tomography^14^, wavelength-tunable lasers^15^, dynamic optical beam splitters^16^, and alignment-free holographic memory systems^17^.

In contrast with other methods of deflecting an optical beam with a KTN crystal, such as using a prism-shaped EO scanner, the space charge-controlled KTN beam deflection has the advantages of simple configuration and a wider scanning angle. In 2008 and 2011, the NTT Photonics Laboratories in Japan proposed two different models to explain the physical mechanisms of the KTN crystal based beam deflection. In the earlier 2008 model^18^, the electrons are instantaneously injected into the crystal by an applied external electric field, which in turn generated a non-uniform electric field that resulted in a non-uniform prism-shaped refractive index distribution via the quadratic EO effect. Such a prism-shaped refractive index distribution could deflect the incoming light beam. In this case, the scanning speed is limited by the electron mobility within the KTN crystal. In the later 2011 model^7^, the electrons are pre-injected and trapped into the KTN crystals. Again, a prism-shaped refractive index distribution is formed via the quadratic EO effect. It was shown that the beam deflection angle was proportional to the product of the pre-injected charge density and the magnitude of the applied external electric field. In this model, the scanning speed is not limited by the electron mobility within the KTN crystal. Although, in theory, the scanning speed could be very fast up to several hundred megahertz^7^, the highest experimentally reported speed was only 700 kHz^19^,^20^.

To figure out the cause of much slower experimental results than theoretical prediction^7^,^20^ and significantly increase the scanning speed of the space charge-controlled KTN beam scanner, we conducted a thorough investigation of the physical mechanism of the electric field-induced phase transition and its influence on the scanning speed of KTN beam scanners. We found that due to the existence of the electric field-induced phase transition at a temperature slightly above the Curie temperature, the KTN crystals operate in the linear EO regime instead of the quadratic EO regime. In this case, the beam deflection angle was only proportional to the injected charge density. To change the beam deflection angle, one has to change the injected charge density, which is limited by the electron mobility in KTN crystals. Thus, the maximum reported scanning speed was only about 700 kHz. To overcome this phase transition-induced speed limitation, we proposed and implemented a space charge-controlled KTN beam scanner not only at a temperature above the Curie point but also at a temperature above the critical end point, which ensured that the KTN scanner operated in the paraelectric phase. We found that there was a three orders-of-magnitude increase in the scanning speed from the microsecond regime to the nanosecond regime by increasing the operational temperature from below the critical end point to above the critical end point. This represents a major technological advance in the speed of optical beam scanners.

## The field-induced phase transition in KTN crystals and its influence on beam deflection

### Field-induced phase transition in KTN crystals

In 2014, T. Imai *et al*.^21^ observed the phenomenon of field-induced phase transition in KLTN crystals. The authors discovered that, even at the temperature above the Curie temperature, the crystal could undergo a phase transition from the paraelectric phase to the ferroelectric phase under an applied external electric field. The higher the electric field and the closer the sample is to the Curie temperature, the more the phase transition is facilitated. Such a transition also occurs in KTN crystals due to their similar crystalline structures.

**Figure 1:**
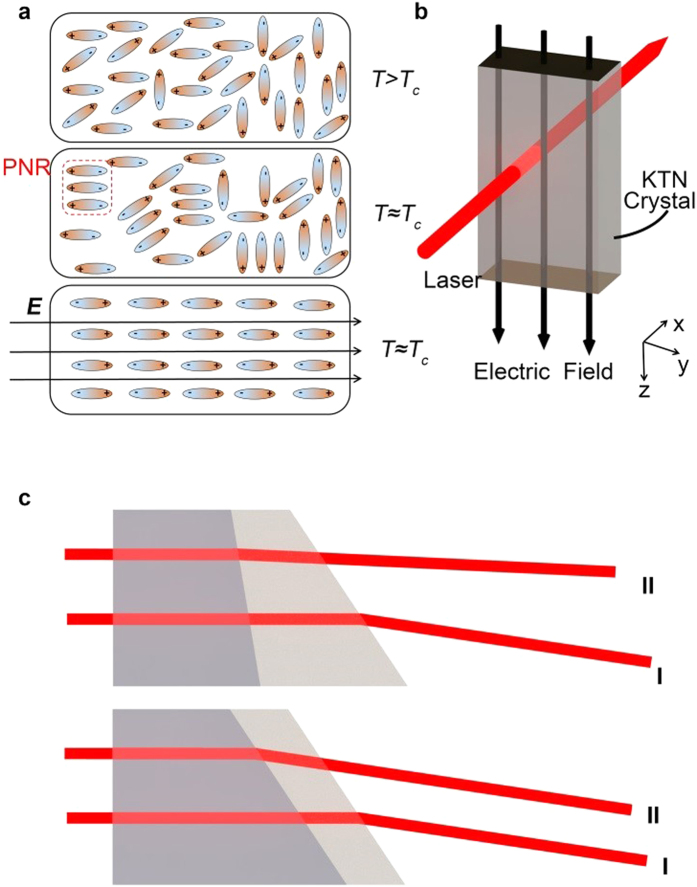
Illustration of a KTN crystal operating under different phases. (**a**) Illustration of the polarization of the KTN under different conditions: at the temperature well above the Curie temperature and with no electric field; at the temperature near the Curie temperature and with no electric field; at the temperature near the Curie temperature and with a high electric field. (**b**) An illustration of a KTN crystal in the ferroelectric phase. (**c**) An illustration of a voltage-controlled variable prism working in the paraelectric phase (upper) and the ferroelectric phase (lower). Beam I represents the light beam passing through the prism without applying an electric field, and Beam II represents the light beam with an applied electric field. In the paraelectric phase, both the thickness and slope of the prism are altered by the applied electric field so that Beam II has a different propagation direction from Beam I. However, in the ferroelectric phase, only the thickness of the prism is altered by the applied electric field, Beam II has a same propagation direction as Beam I, and thus there is no change in the beam deflection angle.

KTN is a solid solution of potassium tantalate (KT) and potassium niobate (KN). It has a Perovskite-type structure^4^. Potassium (K) atoms are located at the corners of each cube and six oxygen (O) atoms are located at the face centers. The niobium (Nb) atoms and tantalum (Ta) atoms are in the center of each cube. When the temperature is well above the Curie temperature, the niobium ions shift randomly from the center of each cube, and hence generate dipoles. Because of the randomness of the dipole directions in each cell, they would cancel each other out, therefore exhibiting a zero polarization at both nanoscopic and macroscopic scales. If the temperature is lowered and maintained near the Curie temperature (but still above the Curie temperature), the thermally-induced random dipole movement becomes weaker. The localized polar nanoregions (PNRs) appear due to the interaction among adjacent dipoles^22^. Although at the nanoscopic scale each PNR has a nonzero polarization, the macroscopic polarization is still zero due to the random polarization orientation of each PNR. The crystal is still in the paraelectric phase. However, if a high electric field is applied on the KTN crystal, the polarization orientation of all the PNRs will be aligned along the direction of external electric field. Consequently, the material has a single polarization direction and operates as if it were in the ferroelectric phase. The three states of KTN crystals described above are as illustrated in Fig. 1(a). Such a field-induced phase transition can be prevented if the temperature is above a certain point, which is called the critical end point^21^.

### Space-charge-controlled KTN beam deflector operating in the paraelectric phase

In the paraelectric phase, based on the 2011 model, the electric field distribution can be expressed as^7^:


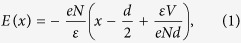


where *V* is the applied voltage, *d* is the electrode gap, *x* is the distance from the cathode, *e* is the elementary electric charge, *ε* is the permittivity, and *N* is the pre-injected and trapped electron charge density. When the temperature is above the critical end point, the field-induced phase transition will not occur, and the KTN crystal operates in the paraelectric phase. Assuming that the polarization of the input light is along the direction of the electric field, the refractive index change induced by the quadratic EO (Kerr) effect is given by^7^:


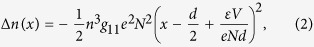


where *n* is the refractive index and *g*_11_ is the quadratic EO coefficient. The corresponding scanning angle is expressed as^7^


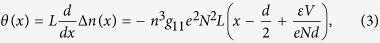


where *L* is the interaction length of the light within the crystal. From the above equation, it is easy to find that the scanning angle is proportional to the product of the applied electric field and the stored electron charge density. If the applied voltage is adjusted, the scanning angle will be altered accordingly. Thus, the scanning speed is only limited by the response time of the voltage source but not the electron migration velocity.

### Space charge-controlled KTN beam deflector operating in the electric field-induced ferroelectric phase

If the temperature is below the critical end point and the applied electric field is large enough, the space charge-controlled KTN beam deflector will operate in the electric field-induced ferroelectric phase. In this case, Eq. (3) has to be modified accordingly. Figure 1(b) illustrates a KTN crystal deflector in the ferroelectric phase. For illustrative purposes, we assume that the direction of the external applied electric field is along the z-axis (i.e., *E*_*x*_ = *E*_*y*_ = 0, *E*_*z*_ ≠ 0) and the field-induced optical axis is also along the z-axis. In this case, *n*_*x*_ = *n*_*y*_ = *n*_*o*_ and *n*_*z*_ = *n*_*e*_, where *n*_*x*_, *n*_*y*_, *n*_*z*_ denote the refractive indices of the crystal for the x-, y-, and z- polarized light, respectively; *n*_*o*_ and *n*_*e*_ are the refractive indices of ordinary ray and the extraordinary ray, respectively.

The principal refractive indices under the electric field are given by (see supplementary information)


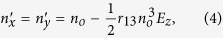



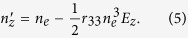


Assuming z-polarized light propagates in this crystal, the refractive index modulation is given by


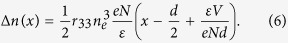


The scanning angle is


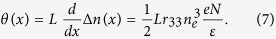


From Eq. (7), it can be seen that the beam deflection angle only depends on the stored charge density. It does not depend on the magnitude of applied electric field. Thus, to change the deflection angle, one has to change the stored charge density. In this case, even with the pre-injected and trapped charges, the scanning speed is still limited by the electron mobility.

As an intuitive illustration, an electro-optic beam deflector can be considered as a voltage-controlled variable prism. As shown in Fig. 1(c), the slope of the output surface and the thickness of the prism can be adjusted by the control voltage in such a variable prism. If the beam deflector operates in the paraelectric phase, both the thickness and slope of the prism changes as the electric field changes. Thus, it can deflect the incoming light beam as the electric field changes. However, if the beam deflector works in the ferroelectric phase, only the thickness of the prism changes as the electric field changes. The slope remains the same, thus it cannot deflect the incoming light beam as the electric field changes. In this case, one has to change the charge density in order to deflect the incoming light beam.

## Results and Discussion

To validate the proposed idea, we fabricated a KTN beam deflector to the following specifications. First, a KTN crystal (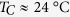) was diced and polished into the following dimensions: 7 mm × 4 mm × 2 mm. Then, the 4 mm × 2 mm top and bottom surfaces were coated with Ti/Au electrodes to facilitate electron injection. After the coating process, the KTN crystal was mounted on a temperature controller and connected to a switchable high voltage power supply. The voltage can be quickly turned on/off within 1–2 ns. A high speed photodetector (FEMTO Messtechnik GmbH HCA-S-200M-Si) was used to measure the response time of the beam deflector.

**Figure 2:**
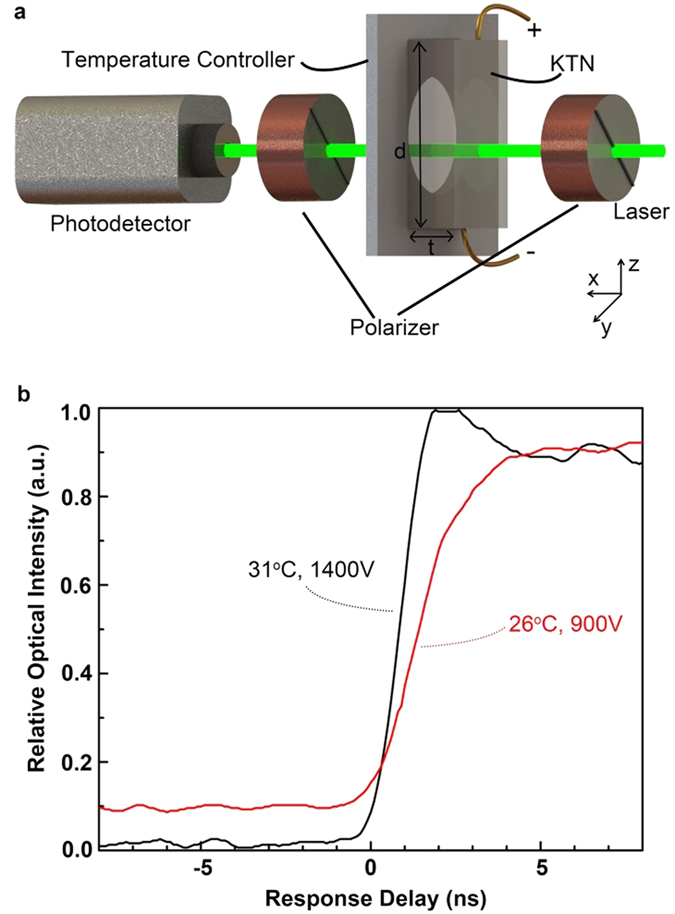
The experimental setup and results for KTN crystal-based switchable half-wave plate. (**a**) An illustration of an EO KTN crystal based switchable half-wave plate. (**b**) The experimentally measured response time of the refractive index change of the KTN crystal as a function of the applied electric field at 26 °C (red) and 31 °C (black).

We first conducted an experiment to determine the response time of the refractive index change as a function of the applied electric field. In the experiment, the EO KTN crystal, which served as an electric field-controlled switchable half-wave plate, was positioned between two 45 degree polarizers, as illustrated in Fig. 2(a). A 532 nm diode-pumped solid-state (DPSS) laser (Coherent, Inc. Compass 532–200) was used as the light source.

When the KTN crystal is in the paraelectric phase, the electric field induced refractive index modulation is expressed as^9^


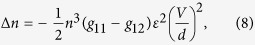


where *n* is the refractive index, *g*_11_ and *g*_12_ are the quadratic EO coefficients for vertical and horizontal polarized light, *ε* is the permittivity, *V* is the applied voltage, and *d* is the electrode gap spacing. The phase difference between the vertical and horizontal polarized light is


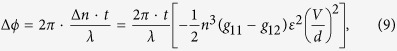


where *t* is the thickness of the KTN crystal, and *λ* is the wavelength of the input light. Then, the quadratic half-wave voltage, 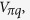 is derived as


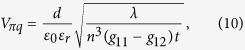


where *λ* is the light wavelength, *ε*_0_ is the vacuum permittivity, and *ε*_*r*_ is the relative permittivity. Without applying the half-wave voltage, there is no change in the polarization direction of the incoming laser beam so that the light can pass through the second polarizer. Upon applying the half-wave voltage, the polarization direction of the incoming light beam is rotated by 90 degrees, which will in turn be blocked by the second polarizer. Thus, when a half-wave voltage is applied on the KTN crystal and then quickly turned off, one can observe a quick increase in the output light intensity.

Similarly, when the KTN crystal is in the ferroelectric phase, based on Eqs (4) and (5), the electric field induced refractive index difference between the vertical and horizontal polarized light is expressed as


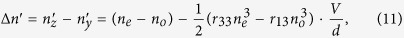


and the corresponding phase difference between the vertical and horizontal polarized light is


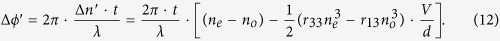


Then, the linear half-wave voltage, *V*_*πl*_, is derived as,


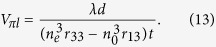


Again, when a half-wave voltage is applied on the KTN crystal and then quickly turned off, one can observe a quick increase in the output light intensity.

In the experiment, when a 900 V voltage was applied on the KTN crystal at 26 °C (near the Curie temperature), we found that the output light intensity reached a minimum value. In other words, the polarization direction of the incoming light was rotated by 90 degrees. When the applied 900 V voltage was quickly switched off, we observed a quick increase in the output light intensity, as shown in Fig. 2(b). The response time was around 3 ns. When a 1400 V voltage was applied on the KTN crystal at 31 °C (well above the Curie temperature), it was found out that the output light intensity reached the minimum. When the applied 1400 V voltage was quickly switched off, one could again observe a quick increase in the output light intensity, as shown in Fig. 2(b). The response time was a little bit faster around 2 ns. At any rate, the response time of the refractive index change of the KTN crystal as a function of the applied electric field was on the order of a nanosecond in both cases. We believe that the response time was mainly limited by the RC time constant of the circuit as well as the response time of the switchable high voltage source itself.

**Figure 3:**
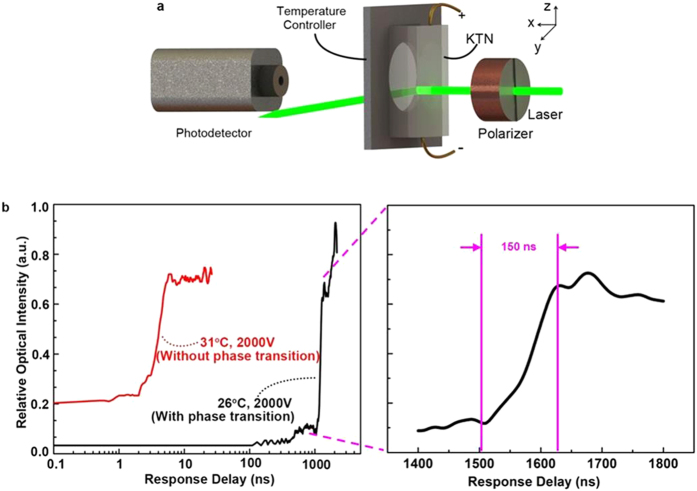
The experimental setup and results for the space-charge-controlled KTN beam deflector. (**a**) An illustration of the experimental setup used to measure the response time of the space charge-controlled KTN beam deflector. (**b**) The experimentally measured response time of the space charge-controlled KTN beam deflector at 26 °C (near the Curie temperature, the electric field-induced phase transition occurred) and 31 °C (well above the Curie temperature, with no electric field induced phase transition in this case).

Figure 3(a) illustrates the experimental setup used to measure the response time of the space-charge-controlled KTN beam deflector. In the system, a polarizer was placed in front of the KTN beam deflector to turn the polarization of the input light in the same direction as the direction of the electric field to maximize the EO effect. Without applying the voltage, the propagation direction of the input light was unaffected by the beam deflector. The input light could reach the photodetector and generated the maximum output signal. When a driving voltage was applied on the KTN crystal, the light beam would be deflected from the photodetector so that one could observe a dramatic drop in the output signal of the photodetector. Or, if a high voltage was applied on the KTN beam scanner and quickly (on the order of a nanosecond) switched off, one would observe a light intensity increase from low level to high level at the nanosecond scale if the response time of the beam deflector was fast enough. Otherwise, it meant that the beam deflector could not follow the high-speed voltage modulation.

In the first experiment, a 2000 V voltage was applied on the KTN beam deflector and then quickly turned off at the temperature 26 °C (near the Curie temperature). Figure 3(b) showed the measured light intensity as a function of time. We observed that the response time was around 1500 ns, which was three orders-of-magnitude longer than the response time of the refractive index change. This experimental result confirmed that the response time of the beam deflection was much longer than that of the refractive index change due to the electric field-induced phase transition. In other words, although there was a refractive index change, the gradient of the refractive index change remained the same at the nanosecond scale, as illustrated in Fig. 1(c). The gradient of the refractive index change only occurred when the charge density was changed, which happened at the microsecond scale due to the limited electron mobility within the KTN crystal. From Fig. 3(b), we observed that the light intensity started to increase quickly after 1500 ns. We speculate that as the electric field was removed, the KTN crystal underwent a phase transition from the field-induced ferroelectric phase back to the paraelectric phase. After such a transition, the scanning speed could increase in the paraelectric phase according to the above analysis. The response time (~1500 ns) in our experiment also matched the previously reported scanning speed^7^,^19^, which was on the order of hundreds of kilohertz.

In the second experiment, a 2000 V voltage was applied on the KTN beam deflector and then quickly turned off at the temperature 31 °C (above critical end point). Figure 3(b) also showed the measured light intensity as a function of time. We observed that the response time was on the order of a nanosecond, which was compatible with the response time of the refractive index change. This experimental result confirmed that the response time of space-charge-controlled KTN beam deflector could be on the order of a nanosecond as long as it operated in the paraelectric phase.

**Figure 4:**
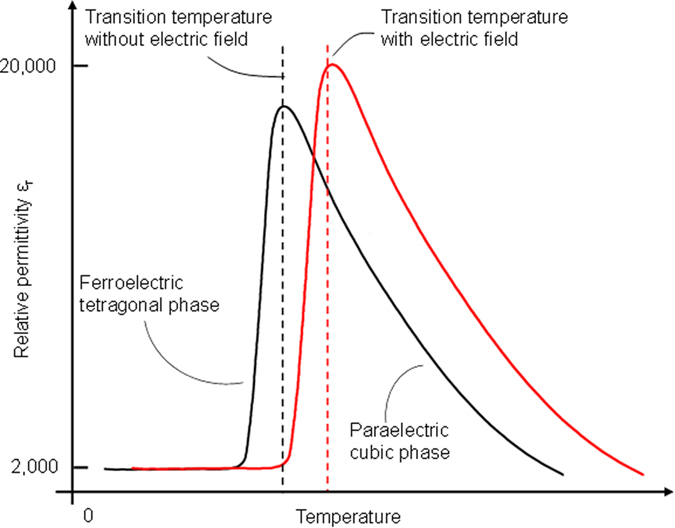
A conceptual illustration of relative permittivity, *ε*_*r*_, as a function of temperature with and without bias electric fields. The electric field shifts the cubic-tetragonal transition to a higher temperature.

To have a better understanding of the above experimental results, we conducted the following analyses. First, we analyzed the quadratic EO effect of the KTN crystal in the ferroelectric phase. Since the relative permittivity, *ε*_*r*_, was small in the ferroelectric phase and the refractive index change induced by the quadratic EO effect was proportional to the square of *ε*_*r*_, the quadratic EO effect of KTN in the ferroelectric phase was substantially smaller than the linear EO effect in the ferroelectric phase, and could be neglected, as discussed in detail below. Figure 4 showed a conceptual illustration of the relative permittivity, *ε*_*r*_, of KTN as a function of temperature with and without bias electric fields. It could be seen that the relative permittivity was around 2000 in the ferroelectric phase and the relative permittivity could be as large as 17500 in the ferroelectric phase when the operational temperature was near the Curie tempearure^23^. Furthermore, the electric field could shift the paraelectric (cubic) to ferroelectric (tetragonal) transition to a higher temperature^24^. The stronger the applied electric field, the larger the shift observed. For example, with a 100 V/mm electric field, there could be a 3 degree Celsius shift in the phase transition temperature upward^24^. Since the transition temperature was 24 °C without the applied electric field (i.e., the case of KTN sample used in this paper), the transition temperature could become higher than 26 °C when a 100+ V/mm electric field was applied. Thus, with a 100+ V/mm electric field, the KTN crystal could be in the ferroelectric phase if the operational temperature was 26 °C, which had a relative permittivity around 2000. To ensure that the 900 V half-wave voltage, obtained at 26 °C, mainly came from the linear EO effect, we made following analysis. By substituting *n* = 2.314, 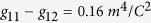, *t* = 2 *mm, λ* = 532 *nm, ε*_0_ = 8.85*x*10^−12^ F/m, *ε*_*r*_ = 2000, and *d* = 7 *mm* into Eq. (10), we obtain the theoretically calculated quadratic half-wave voltage, *V*_*qπ*−*T*_  = 5479 V, which was much higher than the experimentally measured 900 V half-wave voltage. On the other hand, since *n*_0_ ≈ *n*_*e*_ ≈ 2.314 and *r*_33_ ≫ *r*_13_, by substituting *V*_*πl*_ = 900 *V, t* = 2 *mm, λ* = 532 *nm, n*_*e*_ = 2.314, and *d* = 7 *mm* into Eq. (13), we obtain the effective linear EO coefficient 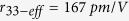 in this field-induced ferroelectric phase. This result made sense because it was less than the peak value of *r*_33_ measured in the pure ferroelectric phase^25^ (i.e., the operational temperature is lower than the original Curie temperature) but was still within the range. In other words, unlike the pure ferroelectric phase, not all PNRs were perfectly aligned in the direction of external electric field at this field induced ferroelectric phase. Thus, the effective *r*_33−*eff*_ was less than the peak value of *r*_33_. Thus, we need only consider the linear EO effect in the regime of EO modulation in the field-induced ferroelectric phase.

Furthermore, since the beam deflection induced by the quadratic EO effect was also very small in the field-induced ferroelectric phase, it could also be neglected as discussed below. Based on Eqs (3) and (7), when light beam passed through the center of the electrode gap (i.e., *x* = *d*/*2*), the amount of beam deflection in the ferroelectric phase, *θ*_*f*_, could be written as


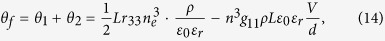


where *ρ* = *e* · *N* was the injected charge density. The first term of Eq. (14), *θ*_1_, represented the contribution from the linear electro-optic effect and the second term of Eq. (14), *θ*_2_, represented the contribution from the quadratic electro-optic effect. As a numerical example, we conducted the following estimation. The static state injected charge density, *ρ*, depended on the magnitude of injecting electric field^26^. When 900 V half-wave voltage was applied, the magnitude of electric field was 900 V/7 mm = 129 V/mm. According to ref. 26, the injected charge density was 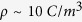 when the penetration depth was larger than 0.5 mm, which was the case in our experiment. By substituting *L* = 2 *mm, r*_33−*eff*_  =  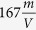, *n*_*e*_ = 2.314, 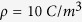, *ε*_0_ = 8.85*x*10^−12^ F/m, and *ε*_*r*_ = 2000 into Eq. (14), we obtain 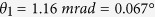. Similarly, by substituting *n* = 2.314, 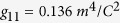, 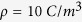, *L* = 2 *mm, ε*_0_ = 8.85*x*10^−12^ F/m, *ε*_*r*_ = 2000, *V*_*π*_ = 900 V, and *d* = 7 *mm* into Eq. (14), we obtain |*θ*_2_| = 0.077 *mrad* = 0.004°, which was 17 times smaller than *θ*_1_. Thus, the contribution to beam deflection from the quadratic EO effect could be neglected in the ferroelectric phase when the 900 V half-wave voltage was applied.

Second, due to the small beam deflection angle in the optical modulation experiment, the effect of beam deflection could be excluded in the optical modulation experiment. As described above, at 26 °C, the 900 V half-wave voltage-induced transversal shift at the focal plane of the photodetector was 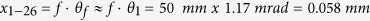 where *f* = 50 mm is the focal length of the lens in front of the photodetector, which was much smaller than the radius of the photodetector (i.e., 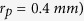. Thus, even if there was beam deflection by applying 900 V, the deflected beam could still be detected as long as the light beam focused on the center of the photodetector when no voltage was applied.

Similarly, At 31 °C, the operational temperature was above the critical end point and the crystal was in the paraelectric phase. In this case, only the second term of Eq. (14) remained, as given by


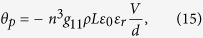


When 1400 V half-wave voltage was applied, the magnitude of electric field was 1400 V/7 mm = 200 V/mm, which increased the injected charge density to the level around 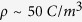. Furthermore, by substituting 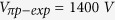 (i.e., the experimentally measured half-wave voltage at 31 °C), *n* = 2.314, 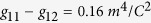, *t* = 2 *mm*, 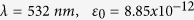 F/m, and *d* = 7 *mm* into Eq. (10), the relative permittivity of our KTN sample at 31 °C was derived as *ε*_*r*_ = 7800. By substituting *n* = 2.314, 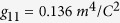, 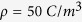, *L* = 2 *mm, ε*_0_ = 8.85*x*10^−12^ F/m, *ε*_*r*_ = 7800, *V*_*π*_ = 1400 V, and *d* = 7 *mm* into Eq. (15), we obtain 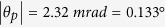. The corresponding amount of transversal shift at the focal plane of the photodetector was 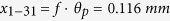, which was again smaller than the radius of the photodetector (i.e., *r*_*p*_ = 0.4 *mm*). Thus, by focusing the light beam on the center of the photodetector when no voltage was applied, the deflected light beam induced by the half-wave voltage could still be detected by the photodetector. Therefore, the influence of beam deflection could be excluded in the EO modulation experiment.

However, to observe the beam deflection effect in the EO deflector, in addition to removing the second polarizer, we made following modifications (although the same sample and geometry were used). First, a higher voltage (~2000 V) was applied at both 26 °C and 31 °C, which could result in a larger deflection due to the increased bias field as well as injected charge density. When a 2000 V was applied, the magnitude of electric field was 2000 V/7 mm = 286 V/mm, which resulted in an even higher injected charge density 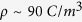.

At 26 °C, the KTN crystal was in the field-induced ferroelectric phase. By substituting *L* = 2 *mm*, 
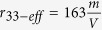
, ne = 2.314, 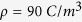, *ε*_0_ = 8.85*x*10^−12^ F/m, and *ε*_*r*_ = 2000 into Eq. (14), one obtained 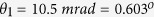. Similarly, substituting *n* = 2.314, 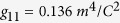, 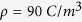, *L* = 2 mm, *ε*_0_ = 8.85*x*10^−12^ F/m, *ε*_*r*_ = 2000, *V* = 2000V, and *d* = 7*mm* into Eq. (12), we obtained 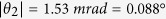, which was still significantly smaller than *θ*_1_ = 0.603°. Thus, the contribution to beam deflection from the quadratic EO effect could again be neglected in the ferroelectric phase even when 2000 V was applied. The corresponding transversal shift became 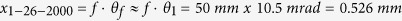, which was larger than the radius of the photodetector (i.e., 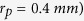. Thus, by focusing the light beam on the center of the photodetector when no voltage was applied, the light beam could be easily deflected away from the photodetector by applying a 2000 V voltage so that the beam deflection effect could be observed.

At 31 °C, the KTN crystal was in the paraelectric phase. Substituting *n* = 2.314, 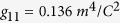, 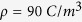, *L* = 2 *mm, ε*_0_ = 8.85*x*10^−12^ F/m, *ε*_*r*_ = 7800, *V* = 2000 V, and *d* = 7*mm*) into Eq. (15), we obtained 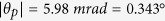. The corresponding transversal shift became 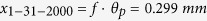 at 31 °C. Although this value (i.e., 0.299 mm) was significantly larger than the one obtained by applying 1400 V (i.e., 0.116 mm), it was still a little bit smaller than the radius of photodetector (0.4 mm). To observe the beam deflection effect, we made a second modification. Instead of focusing the light beam on the center of the photodetector when no voltage was applied, we focused the light beam a little bit off the center of the photodetector in the regime of EO deflector, which ensured that majority of light beam could be deflected away from the photodetector by applying a 2000 V voltage. Unfortunately, since the focused light beam had a Gaussian profile, a small percent of the light beam (i.e., the tail portion of the light beam) was still not totally deflected away from the photodetector even with a 2000 V applied voltage in the paraelectric phase. This was the reason why the initial light intensity was higher (~0.2 a.u.) before 2000 V was switched off for the KTN in the paraelectric phase. Furthermore, although the initial light intensity was much lower at 26 °C than the one at 31 °C in the EO deflector regime due to the larger deflection angle, it was still not zero. Similar to the case of Fig. 2b, this non-zero initial light intensity at 26 °C was caused by the noticeable light scattering of the KTN crystal at 26 °C because it was near the phase transition temperature. Since scattered light propagated at all directions, a small portion of scattered light was detected by the photodetector.

Third, we analyzed the variations in the peak-to-peak voltage between the ON and OFF states for the cases of 26 °C and 31 °C, respectively, which had different causes. In the case of Fig. 2b, the variation in the peak to peak voltage was caused by the difference in light scattering at different temperatures. The closer the sample was to the Curie temperature, the larger the scattering would be because of the increased size of the polar nanoregions (PNR) at the temperature near the Curie temperature. In our experiment, we observed a larger scattering at 26 °C because it was closer to the Curie temperature. Due to the existence of scattering at 26 °C, the linearly polarized light was partially (a small percentage) depolarized after passing through the KTN crystal. Since the depolarized light could not be totally blocked by the second polarizer (i.e., the analyzer), a portion of depolarized light was detected by the photodetector even with half-wave voltage ON. On the other hand, the scattering was much smaller at 31 °C because it was further away from the Curie temperature. In this case, although more light was transmitted after passing through the KTN crystal due to the increased on-line transmittance of KTN crystal, the transmitted light still maintained as the linearly polarized light and could be totally blocked by the second polarizer before reaching photodetector. Therefore, the initial light intensity at 26 °C (i.e., the red curve of Fig. 2b) was higher than the one at 31 °C (i.e., the black curve of Fig. 2b).

In the case of Fig. 3b, the variation in the peak-to-peak voltage was caused by the difference in deflection angle at different temperatures. As described above, the beam deflection-induced transversal shift was 0.526 mm at 26 °C when a 2000 V voltage was applied, which was larger than the radius of the photodetector (i.e., 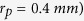. Thus, by focusing the light beam on the center of the photodetector when no voltage was applied, the light beam could be easily deflected away from the photodetector when a 2000 V voltage was applied. This resulted in a lower initial light intensity before 2000 V was switched off for KTN in the ferroelectric phase. However, the beam deflection-induced transversal shift was 0.299 mm at 31 °C when 2000 V was applied, which was a little bit smaller than the radius of photodetector (0.4 mm). Although the beam deflection effect could be observed by focusing the light beam a little bit off the center of the photodetector in the regime of EO deflector, a small percentage of the light beam (i.e., the tail portion of the Gaussian light beam) was still not totally deflected away from the photodetector even with a 2000 V applied voltage in the paraelectric phase. This was the reason why the initial light intensity was higher (~0.2 a.u.) before 2000 V was switched off for KTN at the paraelectric phase.

## Conclusions

In conclusion, we found that the electric field-induced phase transition plays a significant role in determining the scanning speed of a space charge-controlled KTN beam deflector. In the electric field-induced ferroelectric phase, although there could be an electric field-induced refractive index change, the refractive index change gradient remained constant as long as there was no change in the stored charge density. In this case, the response time of a KTN beam deflector is limited by the electron mobility in the KTN crystal even in the presence of the pre-injected and trapped electrons. Thus, for very high-speed nanosecond scale applications, one must avoid the electric field-induced ferroelectric phase by operating the KTN beam deflector at a temperature not only above the Curie point but also above the critical end point. This results in a three orders-of-magnitude increase in the scanning speed from the microsecond to the nanosecond regime; this represents a major technological advance in the field of high speed beam scanning devices. This improvement can be very useful for many applications such as high speed optical coherence tomography, ultrafast reconfiguration free-space optical communications, and ultrafast laser display and printing.

## Additional Information

**How to cite this article**: Zhu, W. *et al*. Three order increase in scanning speed of space charge-controlled KTN deflector by eliminating electric field induced phase transition in nanodisordered KTN. *Sci. Rep.* **6**, 33143; doi: 10.1038/srep33143 (2016).

## Supplementary Material

Supplementary Information
