# Supplementary material for: Three order increase in scanning speed of space charge-controlled KTN deflector by eliminating electric field induced phase transition in nanodisordered KTN
Source: Sci Rep. 2016 Sep 9;6:33143. doi: 10.1038/srep33143 (PMC5017212; doi:10.1038/srep33143)
Supplement: Supplementary Information [file srep33143-s1.pdf]

Three order increase in scanning speed of space-charge-controlled KTN deflector by eliminating electric field induced phase transition in nanodisordered KTN

Supplementary Information

Wenbin Zhu[1], Ju-Hung Chao[1], Chang-Jiang Chen[1], Shizhuo Yin[1,a)], and Robert C. Hoffman[2]

[1]Department of Electric Engineering, Penn State University, University Park, PA 16802.

[2]Robert C. Hoffman, Army Research Lab, 2800 Powder Mill Rd., Adelphi, MD 20783.

a) Corresponding author: sxy105@psu.edu

The Deflection angle of the KTN beam deflector at the ferroelectric phase

The general equation of the index ellipsoid of the linear electro-optic effect can be written as

$$x^2\left(\frac{1}{n_x^2}+r_{11}E_x+r_{12}E_y+r_{13}E_z\right)+y^2\left(\frac{1}{n_y^2}+r_{21}E_x+r_{22}E_y+r_{23}E_z\right)$$
$$+z^2\left(\frac{1}{n_z^2}+r_{31}E_x+r_{32}E_y+r_{33}E_z\right)+2yz\left(r_{41}E_x+r_{42}E_y+r_{43}E_z\right) \quad (1)$$
$$+2xz\left(r_{51}E_x+r_{52}E_y+r_{53}E_z\right)+2xy\left(r_{61}E_x+r_{62}E_y+r_{63}E_z\right)=1,$$

where $\vec{E}=E_x\hat{x}+E_y\hat{y}+E_z\hat{z}$ is the applied external electric field. Since KTN has a 4mm symmetry in its ferroelectric phase, its linear electro-optic coefficients, r_{ij}, are in the form of

$$r_{ij}=\begin{bmatrix} 0 & 0 & \mathrm{r}_{31} \\ 0 & 0 & \mathrm{r}_{31} \\ 0 & 0 & \mathrm{r}_{33} \\ 0 & \mathrm{r}_{51} & 0 \\ \mathrm{r}_{51} & 0 & 0 \\ 0 & 0 & 0 \end{bmatrix}. \quad (2)$$

Substituting Eq. (2) and $n_x=n_y=n_o,\ n_z=n_e$ into Eq. (1), it can be simplified as

$$\left(\frac{1}{n_o^2}+r_{13}E_z\right)x^2+\left(\frac{1}{n_o^2}+r_{13}E_z\right)y^2+\left(\frac{1}{n_e^2}+r_{33}E_z\right)z^2+2r_{51}E_yyz+2r_{51}E_xxz=1. \quad (3)$$

Since the electric field is along the z-axis ($E_x=E_y=0,\ E_z\neq 0$), Eq. (3) can be further simplified as

$$\left(\frac{1}{n_o^2}+r_{13}E_z\right)x^2+\left(\frac{1}{n_o^2}+r_{13}E_z\right)y^2+\left(\frac{1}{n_e^2}+r_{33}E_z\right)z^2=1. \quad (4)$$

The principal refractive indices under the electric field can be given by

$$\frac{1}{n_x'^2}=\frac{1}{n_o^2}+r_{13}E_z,$$
$$\frac{1}{n_y'^2}=\frac{1}{n_o^2}+r_{13}E_z, \quad (5)$$

$$\frac{1}{{n'_z}^2} = \frac{1}{n_e^2} + r_{33}E_z.$$

Since $r_{ij}E \ll n$, Eq. (5) can be further simplified as:

$$n'_x = n'_y \approx n_o - \frac{1}{2}r_{13}n_o^3E_z, \tag{6}$$

$$n'_z \approx n_e - \frac{1}{2}r_{33}n_e^3E_z. \tag{7}$$

Therefore, if a z-polarized light propagates in this crystal, the refractive index modulation is given by

$$\Delta n = -\frac{1}{2}r_{33}n_e^3E_z = \frac{1}{2}r_{33}n_e^3\frac{eN}{\varepsilon}\left(x - \frac{d}{2} + \frac{\varepsilon V}{eNd}\right). \tag{8}$$

The scanning angle is

$$\theta(x) = L\frac{d}{dx}\Delta n(x) = \frac{1}{2}Lr_{33}n_e^3\frac{eN}{\varepsilon}. \tag{9}$$
